# A case of intraductal papillary neoplasm of the bile duct that developed 38 years after choledochoduodenostomy with invasive adenocarcinoma and lymph node metastasis

**DOI:** 10.1186/s40792-019-0651-4

**Published:** 2019-06-07

**Authors:** Mitsuru Kinoshita, Tadafumi Asaoka, Hidetoshi Eguchi, Takehiko Hanaki, Yoshifumi Iwagami, Hirofumi Akita, Takehiro Noda, Kunihito Gotoh, Shogo Kobayashi, Masaki Mori, Yuichiro Doki

**Affiliations:** 1 Department of Gastroenterological Surgery, Graduate School of Medicine, Osaka University, Suita, Japan; 2 Department of Surgery and Science, Graduate School of Medical Sciences, Kyushu University, Fukuoka, Japan

**Keywords:** Intraductal papillary neoplasm of bile duct, Choledochoduodenostomy, Cholangiocarcinoma

## Abstract

**Background:**

Intraductal papillary neoplasm of the bile duct (IPNB) is a bile duct neoplasm characterized as a precursor lesion of cholangiocarcinoma. An invasive component is present in approximately 40 to 80% of reported cases and lymph node metastasis is sometimes detected. We experienced a rare case of IPNB with invasive adenocarcinoma and lymph node metastasis that developed 38 years after choledochoduodenostomy.

**Case presentation:**

A 72-year-old man presented to our hospital for liver dysfunction. The patient had a past medical history of choledochoduodenostomy for a bile duct stone 38 years previously and short bowel syndrome because of strangulation ileus 32 years previously.

Ultrasonography and abdominal enhanced computed tomography (CT) revealed a left intrahepatic bile duct dilation and a papillary mass in the left hepatic duct. Positron emission tomography (PET) CT showed abnormal accumulation in the left hepatic duct and in the hepatic hilar lymph node. Endoscopic retrograde cholangiogram showed a filling defect in the left bile duct, and a cytological examination revealed the presence of atypical cells.

We diagnosed cholangiocarcinoma (derived from IPNB) with lymph node metastasis and performed extended left hepatectomy, caudate lobectomy, and lymph node dissection without extrahepatic bile duct resection.

Histopathological findings showed papillary adenoma and partially invasive poorly differentiated adenocarcinoma in the bile duct. Additionally, the hepatic hilar lymph node was positive.

**Conclusions:**

The tumor was diagnosed as IPNB with invasive adenocarcinoma and lymph node metastasis. Biliary tract cancer that develops after choledochoduodenostomy is extremely rare, and only 17 cases (including IPNB) have been reported in the literature.

## Background

Intraductal papillary neoplasm of the bile duct (IPNB) is a bile duct epithelial tumor with papillosity growth in the bile duct. It is a relatively rare disease that produces a lot of mucus and may cause symptoms of bile duct obstruction (cholangitis or obstructive jaundice) [1]. It is placed as a precancerous or early cancer of the cholangiocarcinoma by the World Health Organization (WHO) Classification of Tumors of the Digestive System 4th edition 2010 and is generally considered to be slow-growing compared with usual cholangiocarcinoma. The prognosis after surgical resection is relatively good [2, 3]. IPNB is a disease concept proposed as a counterpart of intraductal papillary mucinous neoplasm (IPMN). The pathophysiology of IPMN has been clarified in recent years, but much of the pathophysiology of IPNB remains unclear.

Herein, we report a highly suggestive case of IPNB that occurred 38 years after choledochoduodenostomy, with an associated invasive adenocarcinoma and lymph node metastasis.

## Case presentation

A 72-year-old man had been followed up at our hospital, for short bowel syndrome. He had a choledochoduodenostomy for a bile duct stone 38 years prior to this visit and underwent an extensive small intestine excision (residual small intestine, 16 cm) 32 years previously because of strangulation ileus. Therefore, he had required home parenteral nutrition (long-term intravenous hyperalimentation, IVH) for more than 30 years.

He experienced liver dysfunction and presented at our clinical department. He smoked 10 cigarettes/day for 45 years and sometimes drank. His height was 156 cm, he weighed 44.3 kg, and he had a body mass index of 18.3 kg/m^2^.

Physical examination revealed a scar in the midline incision and no tenderness and a palpable mass in the abdomen.

The laboratory data showed the following elevated values: aspartate aminotransferase (AST) 55 IU/L, alanine aminotransferase (ALT) 57 IU/L, lactate dehydrogenase (LDH) 317 IU/L, γGTP 445 IU/L, and alkaline phosphatase (ALP) 1067 IU/L. Tumor markers were slightly elevated, with CEA of 11 ng/ml and CA19-9 of 37 U/ml. Liver infection and hepatitis B and C tests were negative. The other laboratory data were within normal ranges.

**Fig. 1:**
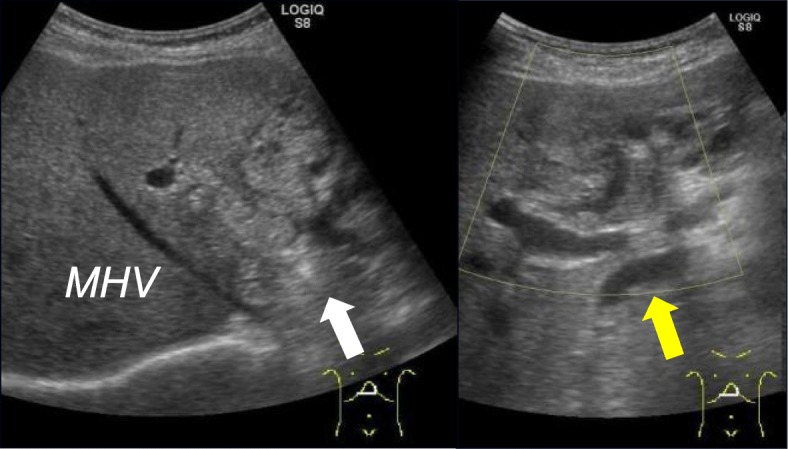
Preoperative ultrasonography. Abdominal ultrasonography showed a hyperechoic lesion, 40 mm in diameter, located in the left hepatic bile duct (white arrow), and extended dilation of the distal left intrahepatic bile duct (yellow arrow)

Abdominal ultrasonography revealed a papillary mass of 40 × 30 mm that was slightly brighter than the surrounding liver tissue in the left hepatic duct, and the distal left intrahepatic bile duct was dilated (Fig. 1).

**Fig. 2:**
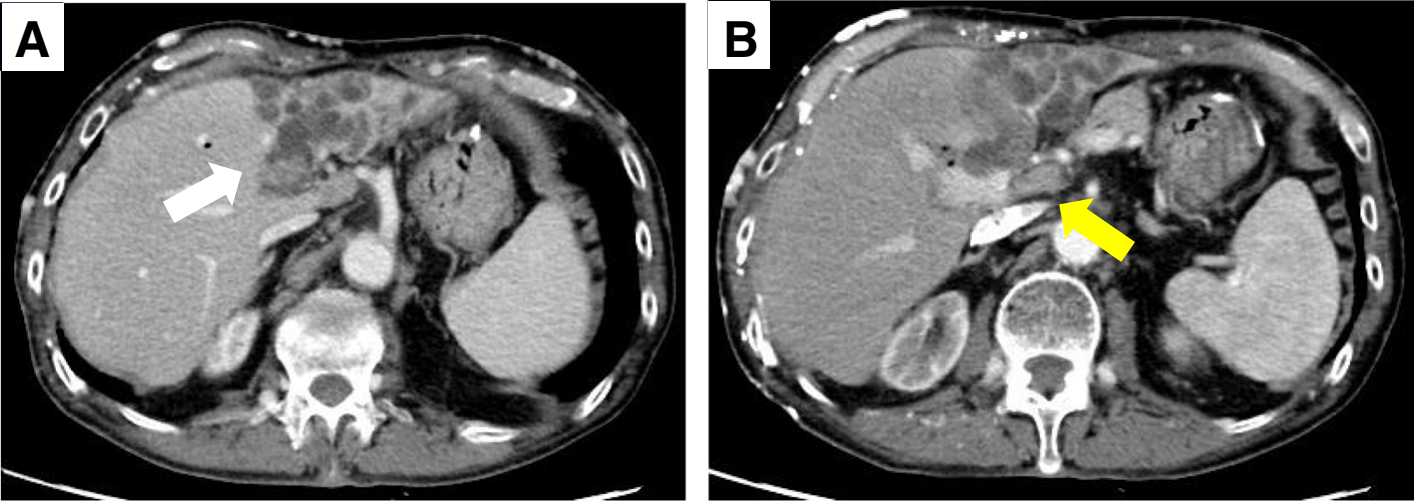
Preoperative enhanced computed tomography. Abdominal axial enhanced computed tomography showed a mass with a rich contrast effect in the left hepatic duct and diffuse dilation of the left intrahepatic bile duct (white arrow) (**a**) and a 12-mm lymph node in the hepatic portal region (yellow arrow) (**b**)

Abdominal enhanced CT also revealed a mass of 40 × 30 mm in the left hepatic duct and a lymph node of 12 mm in the hepatic hilar region (Fig. 2).

**Fig. 3:**
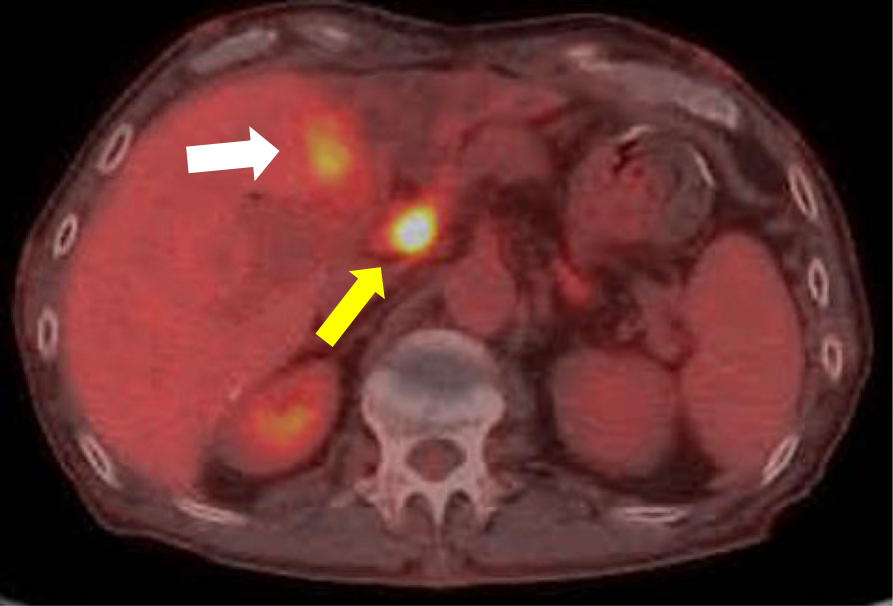
Preoperatively, the ^18^F-fluorodeoxyglucose-positron emission tomography. The F-18 fluorodeoxyglucose-positron emission tomography (FDG-PET) showed abnormal accumulation in the left hepatic duct [the maximum standardized uptake value (SUV max) = 4.6] (white arrow) and in the hepatic hilar lymph node (SUV max = 12.3) (yellow arrow)

The ^18^F-fluorodeoxyglucose-PET (FDG-PET) showed abnormal accumulation in the left bile duct (the maximum standardized uptake value [SUV] max = 4.6) and in the hepatic hilar lymph node (SUV max = 12.3) (Fig. 3).

**Fig. 4:**
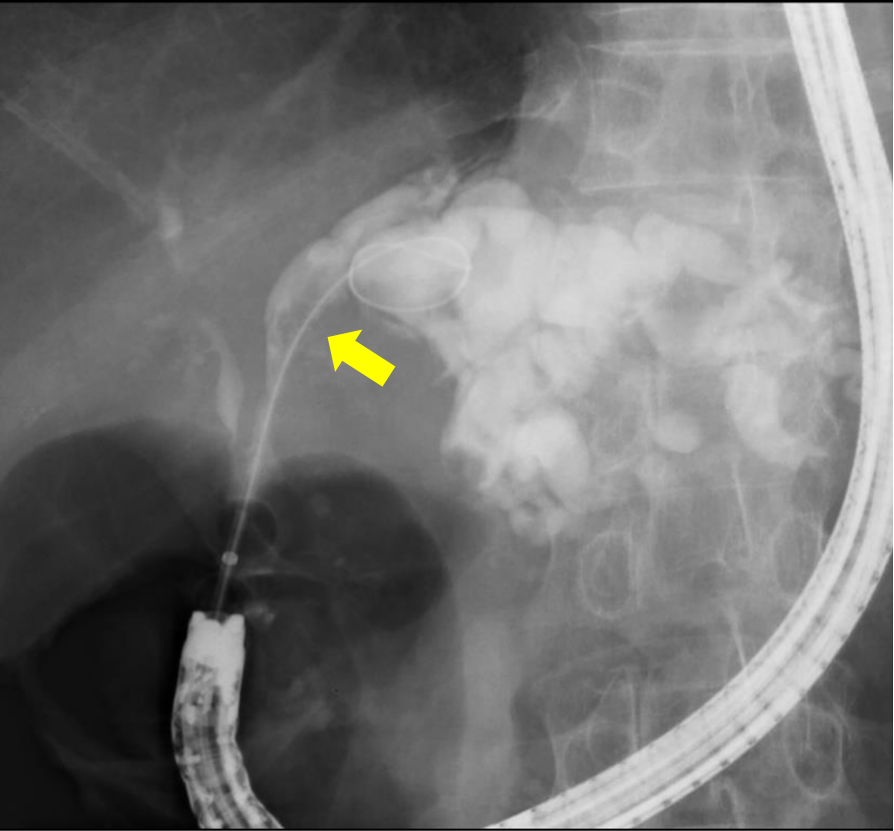
Preoperative endoscopic retrograde cholangiogram (ERC). ERC showed a filling defect in the left bile duct (yellow arrow) and dilation of the left intrahepatic bile duct

Endoscopic retrograde cholangiogram (ERC) showed a filling defect in the left bile duct and dilation of the left intrahepatic bile duct (Fig. 4). However, we could not identify mucus discharge with the endoscope. The bile cytology was class IV, and step biopsy from a root of the left intrahepatic bile duct was negative.

We diagnosed cholangiocarcinoma (derived from IPNB) with lymph node metastasis and performed extended left hepatectomy, caudate lobectomy, and lymph node dissection (lymph node; 8a, 8p, 12a) without bile duct resection.

**Fig. 5:**
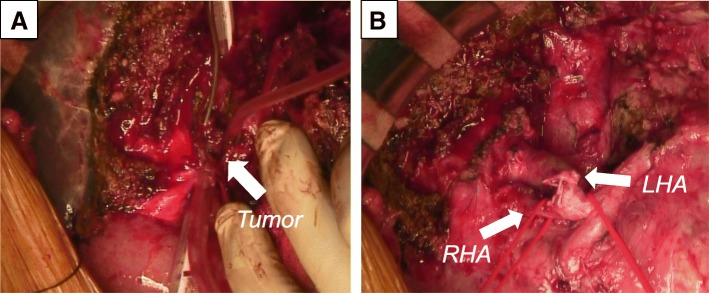
Operative photographs. Intraoperative photographs show a villous tumor in the left bile duct after extended left hepatectomy, caudate lobectomy, and lymph node dissection (**a**) and the bile duct closure after checking the negative of the bile duct stump (**b**)

The operative method was as follows: we confirmed that his liver was green, hard, and elastic using long-term IVH and performed lymph node dissection at first and liver resection without the Pringle maneuver because the postoperative adhesions after choledochoduodenostomy were massive. Moreover, we exposed the left bile duct and removed a villous tumor of the bile duct using an anterior wall incision. We separated a root of the left bile duct, sewed up, and closed after we checked during the surgery that the bile duct stump was negative (Fig. 5).

The reason for this operative strategy was that the patient did not have enough small intestine necessary for a choledochojejunostomy because of his short bowel syndrome.

**Fig. 6:**
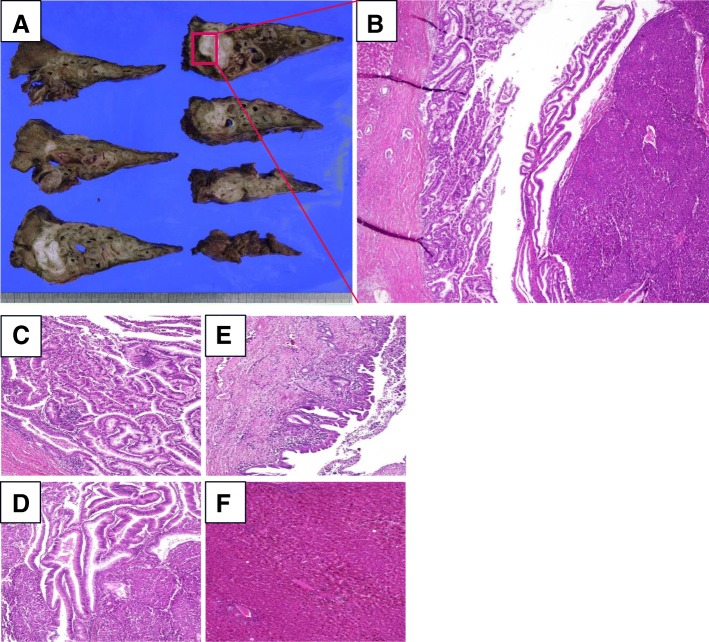
Histopathological findings. Histopathological findings showed a white nodule localized in the bile duct (**a**), papillary adenoma, and poorly differentiated adenocarcinoma (**b**). Transitional zone from adenoma to well-differentiated adenocarcinoma (**c**) and well-differentiated adenocarcinoma to poorly differentiated adenocarcinoma (**d**). There were BilIN around the tumor in the bile duct (**e**), but the liver tissue was normal (**f**). **b** × 40 magnification, **c**–**f** × 100 magnification

Histopathological findings showed papillary adenoma, with well-differentiated nuclear atypia and partially invasive, poorly differentiated adenocarcinoma in the bile duct. There was biliary intraepithelial neoplasia-1 (BilIN-1) around the tumor in the bile duct, and the liver tissue was normal (Fig. 6). The hepatic hilar lymph node was positive.

**Fig. 7:**
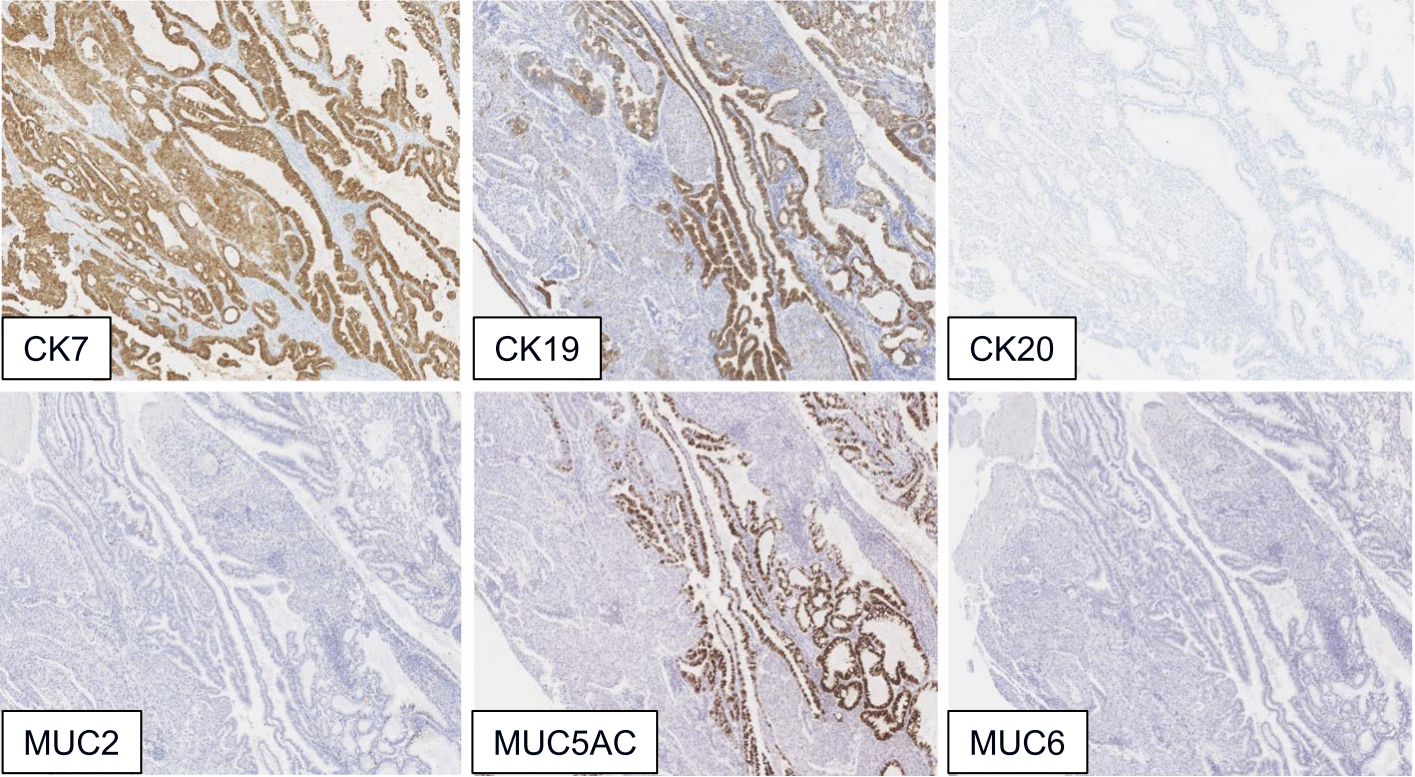
Immunohistochemistry. Immunohistochemistry revealed that CK7, CK19, and MUC5AC are positive in the papillary region.CK20, MUC2, and MUC6 are negative

Immunohistochemistry revealed that CK7, CK19, and MUC5AC were positive in the papillary region (Fig. 7).

We could confirm the continuous change from adenoma to well-differentiated adenocarcinoma and well-differentiated adenocarcinoma to poorly differentiated adenocarcinoma. Thus, we diagnosed cholangiocarcinoma derived from IPNB.

The patient’s postoperative course was good, and he was discharged on the 15th day after surgery. However, he had para-aortic lymph node recurrence 10 months later and has received chemotherapy with gemcitabine.

## Discussion

IPNB is characterized by a dilated intrahepatic bile duct filled with a non-invasive papillary or villous biliary neoplasm covering delicate fibrovascular stalks. It is classified as low, intermediate, or high grade based on the degree of cellular and nuclear atypia. It is considered a precancerous or early cancer of the cholangiocarcinoma by the WHO Classification of Tumors of the Digestive System 4th edition 2010 [2, 3]. Currently, IPNB is considered part of the bile duct adenomas, papillary cholangiocarcinoma, cholangiocarcinoma with intrabiliary growth, and mucin-producing bile duct tumors. It is still controversial as to which classification it mainly belongs to.

The clinical finding of IPNB is generally as follows: the average age of presentation is 60 years, it is more common in men than in women, the location is more often on the left bile duct than on the right, and the symptoms are similar to those for bile duct obstruction (cholangitis or obstructive jaundice). The 5-year survival rate after R0 surgical resection is 59.7%, which is considered to be better than that for intrahepatic cholangiocarcinoma (5-year survival rate is 23.9%) [4–8].

Recently, Japan-Korea experts have proposed the new classification for IPNB [9]. In the manuscript, they classified IPNB to type 1 IPNB and type 2 IPNB. The type 1 IPNB is the IPNB in the narrow sense, showing the typical pathological findings, and being assumed to be the counterpart of intraductal papillary mucinous neoplasm (IPMN) of the pancreas. While the type 2 IPNB shows various pathological presentations, sometime with solid tumor component. Conventional papillary carcinoma belongs to type 2 IPNB. Our case was considered to be applicable to type 2 IPNB because there was a solid component and the relatively thick fibrovascular stalk as shown in Fig. 6.

Histological subclassifications of IPNB have been described for four phenotypes of tumors, similar to IPMN: gastric type, intestinal type, pancreatobiliary type, and oncocytic type as well as IPMN, depending on the appearance of the cell by hematoxylin and eosin staining and the staining pattern of mucin core proteins. The gastric type shows columnar cells with abundant intracytoplasmic mucin, a clear cytoplasm, and MUC5AC on immunohistochemistry is positive. The intestinal type shows stratified tall columnar cells, occasionally with goblet cells, and staining for MUC2 or MUC5AC is positive. The pancreatobiliary type exhibits columnar cells, with an eosinophilic or pale eosinophilic cytoplasm and round nuclei and staining for MUC1 is positive. The oncocytic type has abundant eosinophilic cytoplasm and round nuclei and stains positively for MUC5AC and MUC6 and/or MUC2 and MUC1 [10].

Our case was determined to be the intestinal type based on the presence of columnar cells morphologically and the MUC5AC-positive staining.

IPNB is considered to be caused by cholestasis and biliary tract infection as well as biliary tract cancer. Generally, it is easy to work out the occlusion mechanism in the biliary tract, and it is generally accompanied by a bacterial infection and an inflammatory reaction. Recently, a multistage carcinogenesis to hyperplasia-dysplasia-carcinoma sequence is proposed as a mechanism for biliary tract cancer due to chronic inflammation [11]. Chronic inflammatory conditions induce the production of reactive oxygen or nitrogen species leading to DNA damage, play important roles in cholangiocarcinogenesis [12].

After choledochoenterostomy, as mentioned above, chronic inflammation may occur and is considered to be one of the factors involved in the development of cholangiocarcinoma. The incidence rate of cholangiocarcinoma is estimated at 7.6% after choledochoduodenostomy and at 1.9% after choledochojejunostomy [13]. We examined about 17 patients with biliary tract cancer that occurred after choledochoduodenostomy and that were recognized by the Japan Medical Abstracts Society (1983–2016) (Table 1) [14–25]. The most frequent cancer location was the anastomotic and portal parts. After choledochoduodenostomy, it is possible that carcinogenesis may occur due to the damage and regeneration of the bile duct epithelium after long-term exposure to intestinal fluid and due to physical stimulation because of elevated biliary duct pressure. It is reasonable to think that our case also occurred in the left bile duct for anatomical reasons.

**Table 1:** After choledochoduodenostomy, 17 patients were diagnosed with biliary tract cancer (Japan Medical Abstracts Society; 1983–2016)

Variables	*n* = 17
Gender (male/female)	7 (41%)/10 (59%)
Age (years)	62 (41–73)
Previous history (bile duct)	
Stone	8 (47%)
Dilation	5 (29%)
Injury	4 (24%)
Period (years)	32 (12–40)
Type	
Well	1 (6%)
Moderately	3 (18%)
Poorly	5 (29%)
Papillary	3 (18%)
Unknown	5 (29%)
Location	
Anastomotic	9 (54%)
Portal	3 (18%)
Left hepatic duct	2 (12%)
Right hepatic duct	1 (6%)
S6	1 (6%)
S5	1 (6%)
Operative procedure	
PD	3 (18%)
HPD	2 (12%)
Left lobectomy	2 (12%)
Partial	1 (6%)
Choledochectomy	1 (6%)
Stent	3 (18%)
PTBD	2 (12%)
Bypass	1 (6%)
Microwave	1 (6%)
Unresected	1 (6%)
Outcome	
Alive	8 (47%)
Dead	9 (53%)

*PD* pancreatoduodenectomy, *HPD* hepato-pancreaticoduodenectomy

As for the operative procedure, if the tumor is in the anastomotic site, pancreatoduodenectomy (PD) is performed, and if the tumor is above the anastomotic site, hepatopancreatoduodenectomy (HPD) is performed. All cases in which an R0 resection was possible were alive. In our case, there was also an indication for HPD, but because of the patient’s short bowel syndrome, it was impossible to reconstruct the biliary tract; therefore, we performed an extended left hepatectomy and caudate lobectomy without choledochojejunostomy.

Our report is a highly suggestive case to explore the origin of IPNB.

That is, pathologically, IPNB is the main constituent and poorly differentiated adenocarcinoma and BilIN are in a part of it. A series of multistage carcinogenic events is considered in cases of gradual malignant progression over a long period of time, leading to lymph node metastasis. We reviewed 17 patients with biliary tract cancers (including IPNB) that developed after choledochoduodenostomy. In all cases, the cancer developed more than 10 years after the operation. We should follow patients for the development of biliary tract cancer for long periods after cholangioenterostomy.

## Conclusions

We reported a rare surgical case of IPNB with invasive adenocarcinoma and lymph node metastasis that developed 38 years after choledochoduodenostomy.

It showed interesting histopathological findings in which BilIN, adenoma, and adenocarcinoma are mixed. These can be important indicators in considering the genesis and progression of cancer.

## Abbreviations

ALP: Alkaline phosphatase
ALT: Alanine aminotransferase
AST: Aspartate aminotransferase
BilIN: Biliary intraepithelial neoplasia
CT: Computed tomography
ERC: Endoscopic retrograde cholangiogram
FDG-PET: Fluorodeoxyglucose-positron emission tomography
HPD: Hepatopancreatoduodenectomy
IPNB: Intraductal papillary neoplasm of the bile duct
LDH: Lactate dehydrogenase
PD: Pancreatoduodenectomy
SUV: Standardized uptake value
WHO: World Health Organization
